# Supplementary material for: Thermally activated intermittent dynamics of creeping crack fronts along disordered interfaces
Source: Sci Rep. 2021 Oct 14;11:20418. doi: 10.1038/s41598-021-98556-x (PMC8516960; doi:10.1038/s41598-021-98556-x)
Supplement: Supplementary file 1 — Supplementary Information. [file 41598_2021_98556_MOESM1_ESM.pdf]

# Supplementary Information

# Thermally activated intermittent dynamics of creeping crack fronts along disordered interfaces

**Tom Vincent-Dospital[1,2,*], Alain Cochard[1,*], Stéphane Santucci[3,4], Knut Jørgen Måløy[2], and Renaud Toussaint[1,2,*]**

[1]Université de Strasbourg, ITES UMR 7063, Strasbourg F-67084, France
[2]SFF Porelab, The Njord Centre, Department of physics, University of Oslo, Norway
[3]Université de Lyon, ENS de Lyon, Université Claude Bernard, CNRS, Laboratoire de Physique, France
[4]Lavrentyev Institute of Hydrodynamics, Siberian Branch of the Russian Academy of Sciences, Novosibirsk, Russia
[*]tom.vincent-dospital@fys.uio.no, alain.cochard@unistra.fr and renaud.toussaint@unistra.fr

## 1 On the negligible temperature elevation

In this manuscript, we have considered that the temperature elevation at the crack front $\Delta T$, arising from the release of energy $G$, was negligible. In this section, we discuss this assumption further. To assess $\Delta T$, We use a quasi-static thermal model that we have introduced in several previous works[1–3]. There, cracks having a velocity less than $\lambda/(\pi C l)$ experience a rise in temperature

$$\Delta T \sim \frac{\phi G V}{\lambda}, \tag{1}$$

where $\phi$ is a heat conversion efficiency as the front advances, where $\lambda = 0.18\,\mathrm{J\,s^{-1}\,m^{-1}\,K^{-1}}$ and $C = 1.5\,\mathrm{MJ\,m^{-3}}$ denote, respectively, the heat conductivity and capacity of PMMA[4], and where $l$ is the radius of a heat production zone around the front. In the case of bulk PMMA, it was inverted[3] that $\phi \sim 0.2$ and $l \sim 10\,\mathrm{nm}$. Of course, applying these bulk PMMA thermal parameters to the rupture dynamics of sintered interfacial PMMA is already an assumption, as both are, in practice, two different (although similar) materials. With this assumption, Eq. (1) above is valid for $V < \lambda/(\pi C l) \sim 4\,\mathrm{m\,s^{-1}}$, that is, it is valid for any velocity that we have modelled, which is less than $1\,\mathrm{mm\,s^{-1}}$ (see Fig. 3 of the main manuscript). With this conservative $V = 1\,\mathrm{mm\,s^{-1}}$ and with a maximal load of about $250\,\mathrm{J\,m^{-2}}$ (see Fig. 13a of the main manuscript), the maximal temperature elevation $\Delta T$ computes to less than a degree and, thus, is negligible compared to $T_0 = 298\,\mathrm{K}$. In this context, our adiabatic crack front hypothesis is verified.

## 2 On the physical meaning of the $\alpha$ parameter

The $\alpha^2$ parameter is a particularly small (subatomic) area. This parameter was directly fitted on the creep curve of the studied interfacial PMMA, showing an exponential dependence of the average front velocity with the mean crack energy release rate[5]. In Refs.[3,6,7], we explain how $\alpha$ is not an actual physical size of the rupturing material. Instead, $\alpha^2$ is an equivalent area in the order of $d_0^3/l$, where $d_0$ is the typical intra-molecular distance (called for in a thermally activated context) and where $l$ is the scale limiting the stress divergence – and thus the local storage of energy – at the crack tip. Assuming $d_0$ to be about 0.3 nanometers, one gets $l$ in the order of 1-10 nanometers. This size is orders of magnitude smaller than the typical process zone length around crack tips in PMMA (10 to 100 micrometers). There is, however, no strong reason to consider a Dugdale[8] description of the stress in the process zone, that is, that the stress saturates at scales below 10 to 100 micrometers from the front. It is instead likely that the stress remains an increasing function up to small scales inside the process zone. In PMMA, a few nanometers (i.e., $l$) is a physically reasonable length scale, as it is both the size of a few MMA radicals and a typical length scale for the entanglement density[9] in the polymer.

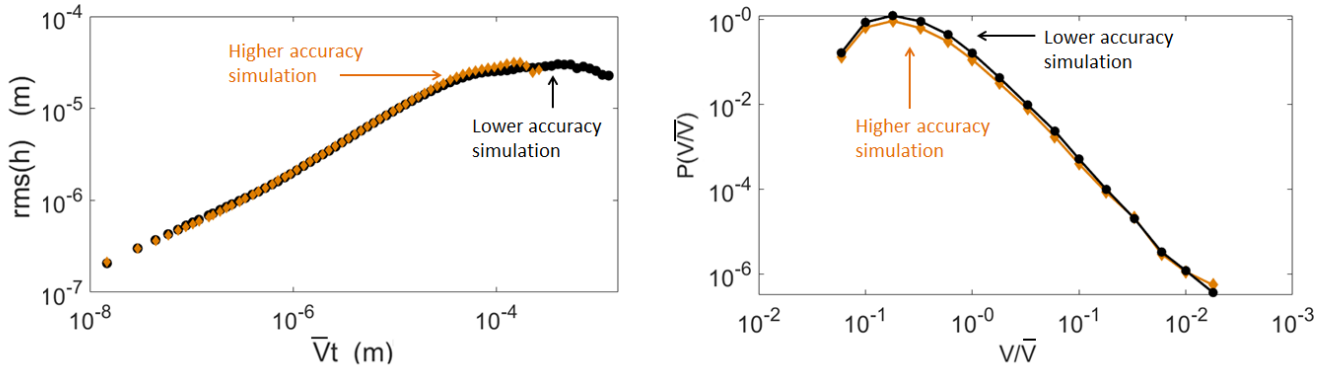


**Figure 1.** Front width growth (a) and local velocity distribution (b) for two simulations with the same physical parameters but different numerical accuracies, as per table 1 below. The points are data from the simulation analysed in this manuscript and the squares were computed on denser numerical grids. The computed exponents are not significantly affected. The most refined (heavier to run) simulation was run on a shorter time span, which explains why $\overline{V}t$ stops at about $2 \times 10^4$ m.

## 3 Solver convergence

We verified that our simulations were accurate enough so that the derived statistical features were not dependent on the steps of the numerical computation grids. In table 1 below, we show the accuracy parameters of two different simulations and show, in Fig. 1, how the modelled crack dynamics is unchanged with both parameter sets.

| Parameter | Higher accuracy | Lower accuracy | Unit |
|---|---|---|---|
| $\Delta x_s$ | 0.5 | 1 | µm |
| $\Delta t_s$ | $\sim 1$ | $\sim 5$ | ms |
| $L_s$ | 12000 | 6000 | µm |

**Table 1.** Two different sets of numerical accuracy parameters corresponding to the results shown in Fig. 1.

## 4 On the time dependency of $\overline{G}$

We have considered $\overline{G}$, in Eq. (2) of the main manuscript, to be a constant when running our simulations. In practice, the mean energy release rate is to vary during the progression of a crack. In the set-up of Tallakstad *et al.*[10] (shown in Fig. 1 of the main manuscript), $\overline{G}$ increases with the lower plate's deflection, while it decreases with the mean advance of the front $\overline{a}$. Using the Euler–Bernoulli beam theory[11], one can actually compute[5] the mean energy release rate at the tip of such a system:

$$\overline{G}(t) = \frac{3Eh_p^3 u(t)^2}{8\overline{a}(t)^4} \quad \text{if } \overline{a} \gg h_p, \tag{2}$$

with $E$ the lower plate Young modulus, $h_p$ its thickness, and $u$ the plate deflection (in meter). Two loading conditions were used in the experiments that we have here reproduced. One corresponds to a forced regime, where $u$ increases linearly with time, and where it was shown[5] that the resulting $\overline{G}$ is rather constant. The other regime is a relaxation one, where $u$ is kept constant while the crack continues to creep. In both cases, the long term evolution of $\overline{G}$ was shown[5,12] to be reproduced by Eqs. (1) and (2) of the main manuscript. In the second (relaxation) regime, $\overline{G}$ decreases with time, by a percentage given by Eq. (2) above: $[\overline{a}_0/(\overline{a}_0 + \Delta\overline{a})]^4$, where $\overline{a}_0$ is the crack length at the beginning of an experiment (typically 10 cm) and $\Delta\overline{a}$ is the total crack advancement during a realisation, which is in the order of 1 mm, similarly to what is shown in Fig. 3 of the main manuscript. The mean energy release rate thus decreases by about 4% during a non-forced experiment, and even less so (0.4%) during typical avalanches of extent less than 0.1 mm (see Fig. 9b of the main manuscript). In comparison, the spacial standard deviation of the energy release, as predicted by Eq. (2) of the main manuscript and as shown here for a given time $t$ in Fig. 2a, accounts for about 30% of $\overline{G}$. The time evolution of $G$ is hence small compared to its spacial variations, and modelling $\overline{G}$ as a constant is thus appropriate to study the burst-like dynamics of the experimental[10] cracks. Note however that it is possible to include the above Eq. (2), or any other mechanical load descriptor, in the numerical solver, as done with our model by Cochard *et al.*[12]. In Fig. 2b of this supplementary information, we show that the velocity distribution does not change significantly when

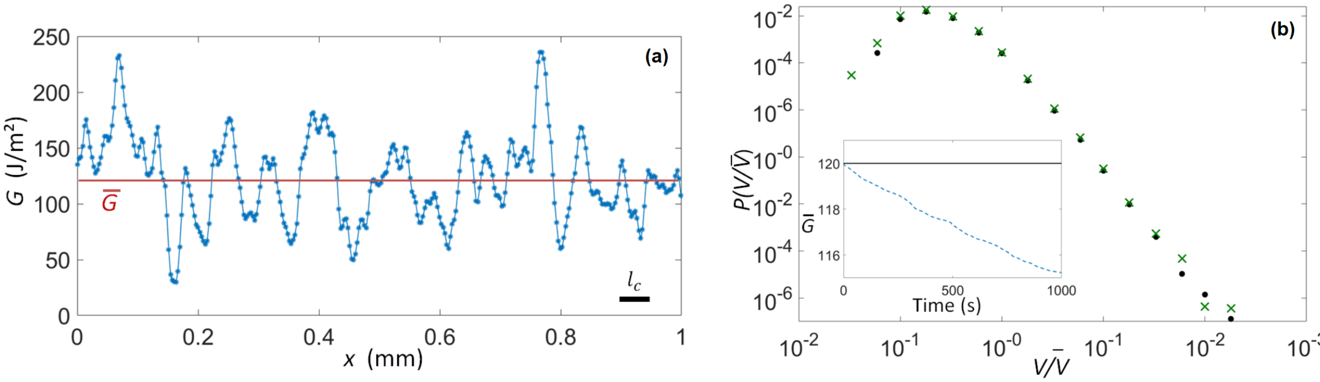


**Figure 2.** (a): Lateral variation of the energy release rate $G$ along a portion of the crack front, at a given simulation time, as predicted by Eq. (2) of the main manuscript and with the parameters of table 1 of this main manuscript. The straight line shows $\overline{G} = 120\,\mathrm{J\,m^{-2}}$. The standard deviation of $G$ computes to about 30% of this average value. For reference, the scale bar shows the correlation length $l_c = 50\,\mu\mathrm{m}$ of the material disorder. (b): Local velocity distribution for a simulation with a constant load $\overline{G}$, as in the manuscript (points), and with a relaxing load as the crack advances (crosses), as per Eq. (1) of the main manuscript. The inset shows the time evolution of $\overline{G}$ for the former (plain line) and the latter (dashed line). The load relaxation during the second simulation stays small if compared to its lateral variation, as shown in (a).

describing a relaxing load as the crack advances over the typical experimental course, compared to the constant $\overline{G}$ case studied in this manuscript.

## 5 Varying the modelled mean velocity

In Fig. 3 below, we show that the intermittent dynamics of the simulated fronts is not strongly dependent on the average propagation velocity of the crack. This is consistent with the experimental observations from Tallakstad *et al.*[10], where many driving velocities were used.

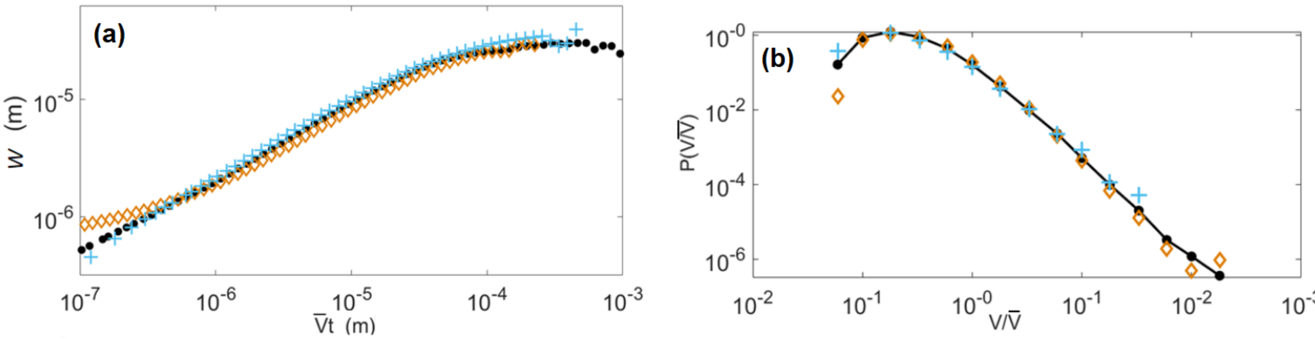


**Figure 3.** Front width growth (a) and local velocity distribution (b) for three simulations with a different average energy release rate: $\overline{G} = 100\,\mathrm{J\,m^{-2}}$ (crosses), $\overline{G} = 120\,\mathrm{J\,m^{-2}}$ (points) and $\overline{G} = 140\,\mathrm{J\,m^{-2}}$ (squares). This corresponds to an average propagation velocity $\overline{V}$ of, respectively, $0.06\,\mu\mathrm{m\,s^{-1}}$, $1.5\,\mu\mathrm{m\,s^{-1}}$ and $25\,\mu\mathrm{m\,s^{-1}}$. Both statistics show little dependency on $\overline{V}$, which is consistent with the experimental results[10].

## References


1. Toussaint, R. *et al.* How cracks are hot and cool: a burning issue for paper. *Soft Matter* **12**, 5563–5571, DOI: 10.1039/C6SM00615A (2016).
2. Vincent-Dospital, T., Toussaint, R., Cochard, A., Måløy, K. J. & Flekkøy, E. G. Thermal weakening of cracks and brittle-ductile transition of matter: A phase model. *Phys. Rev. Mater.* DOI: 10.1103/PhysRevMaterials.4.023604 (2020).
3. Vincent-Dospital, T. *et al.* How heat controls fracture: the thermodynamics of creeping and avalanching cracks. *Soft Matter* DOI: 10.1039/d0sm010 (2020).
4. Technical information, Altuglas sheets. Tech. Rep., Arkema (2017).
5. Lengliné, O. *et al.* Average crack-front velocity during subcritical fracture propagation in a heterogeneous medium. *Phys. Rev. E* **84**, 036104, DOI: 10.1103/PhysRevE.84.036104 (2011).
6. Vanel, L., Ciliberto, S., Cortet, P.-P. & Santucci, S. Time-dependent rupture and slow crack growth: elastic and viscoplastic dynamics. *J. Phys. D: Appl. Phys.* **42**, 214007, DOI: 10.1088/0022-3727/42/21/214007 (2009).
7. Vincent-Dospital, T., Toussaint, R., Cochard, A., Flekkøy, E. G. & Måløy, K. J. Thermal dissipation as both the strength and weakness of matter. a material failure prediction by monitoring creep. *Soft Matter* DOI: 10.1039/D0SM02089C (2021).
8. Dugdale, D. Yielding of steel sheets containing slits. *J. Mech. Phys. Solids* **8**, 100 – 104, DOI: 10.1016/0022-5096(60)90013-2 (1960).
9. Henkee, C. S. & Kramer, E. J. Crazing and shear deformation in crosslinked polystyrene. *J. Polym. Sci. Polym. Phys. Ed.* **22**, 721–737, DOI: 10.1002/pol.1984.180220414 (1984).
10. Tallakstad, K. T., Toussaint, R., Santucci, S., Schmittbuhl, J. & Måløy, K. J. Local dynamics of a randomly pinned crack front during creep and forced propagation: An experimental study. *Phys. Rev. E* **83**, 046108, DOI: 10.1103/PhysRevE.83.046108 (2011).
11. Anderson, T. L. *Fracture Mechanics: Fundamentals and Applications* (Taylor and Francis, 2005).
12. Cochard, A., Lengliné, O., Måløy, K. J. & Toussaint, R. Thermally activated crack fronts propagating in pinning disorder: simultaneous brittle/creep behavior depending on scale. *Philos. Transactions Royal Soc. A : Math. Phys. Eng. Sci.* DOI: 10.1098/rsta.2017.0399 (2018).
